# Supplementary material for: A Multimodal Biomarker Predicts Dissemination of Bronchial Carcinoid
Source: Cancers (Basel). 2022 Jun 30;14(13):3234. doi: 10.3390/cancers14133234 (PMC9265109; doi:10.3390/cancers14133234)
Supplement: Supplementary file 1 [file cancers-14-03234-s001.zip › SupplementaryFigureS1.pdf]

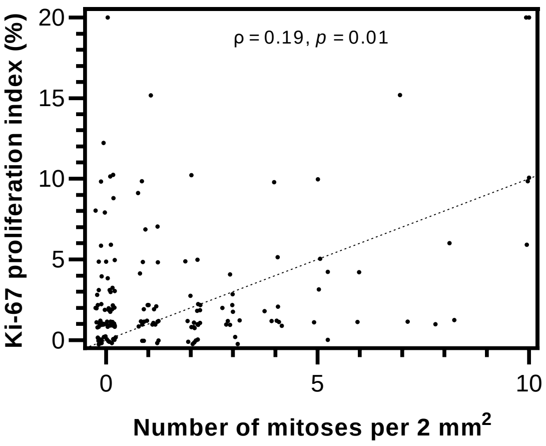

Figure S1. Spearman's rho correlation between Ki-67 proliferation index and number of mitoses per 2 mm^2; ρ: Spearman rank-order correlation coefficient.
