# Supplementary material for: A Multimodal Biomarker Predicts Dissemination of Bronchial Carcinoid
Source: Cancers (Basel). 2022 Jun 30;14(13):3234. doi: 10.3390/cancers14133234 (PMC9265109; doi:10.3390/cancers14133234)
Supplement: Supplementary file 1 [file cancers-14-03234-s001.zip › SupplementaryTableS1.pdf]

Table S1

Antibody	Company	Clone	Species	Reference number	Dilution	IHC incubation time (min)	Detection method
CD044	Ventana	SP37	Rb	06364985001	RTU	32	Optiview
MIB 1	Dako	MIB1	Mo	M7240	1/50	32	Optiview
OTP	Sigma-Aldrich	Polyclonal	Rb	HPA059342	1/50	64	Optiview
RB	BD Biosciences	G3-245	Mo	554136	1/1000*	32	Optiview
p16	Ventana	E6H4	Mo	06695248001	1/2	32	Optiview

Table S1. Characteristics of antibodies used for immunohistochemistry; IHC: immunohistochemistry; RTU: ready-to-use; * in Dako Antibody Diluent.
