# Supplementary material for: A Multimodal Biomarker Predicts Dissemination of Bronchial Carcinoid
Source: Cancers (Basel). 2022 Jun 30;14(13):3234. doi: 10.3390/cancers14133234 (PMC9265109; doi:10.3390/cancers14133234)
Supplement: Supplementary file 1 [file cancers-14-03234-s001.zip › SupplementaryTableS2.pdf]

Marker	Number of IHC positive cases (%)[∞]			Median H-score / Ki-67 score (SD)		
	TC	AC	*p*-value[§]	TC	AC	*p*-value[a]
Rb	112/112 (100)	59/59 (100)	*NA*	180 (77)	200 (82)	0.102
p16	30/111[*] (27)	21/59 (36)	0.246	0 (32)	0 (49)	0.126
OTP	96/112 (86)	45/59 (76)	0.123	240 (106)	300 (126)	0.608
CD44	96/111[*] (86)	43/58[*] (74)	0.046	300 (104)	300 (134)	0.055
Ki-67	*NA*	*NA*	*NA*	1 (3)	2 (4)	0.134

Table S2. Immunohistochemistry results for TC and AC; IHC: immunohistochemistry; TC: typical carcinoid; AC: atypical carcinoid; * one missing p16 or CD44 value; ∞ excluding three cases with missing mitotic figures; § calculated using the Chi-Squared test; [a] calculated using the Mann-Whitney U test; significant *p*-values are reported in bold.
