# Supplementary material for: A Multimodal Biomarker Predicts Dissemination of Bronchial Carcinoid
Source: Cancers (Basel). 2022 Jun 30;14(13):3234. doi: 10.3390/cancers14133234 (PMC9265109; doi:10.3390/cancers14133234)
Supplement: Supplementary file 1 [file cancers-14-03234-s001.zip › SupplementaryTableS3.pdf]

| Variable | Total (%) | TC (%) | AC (%) | *p*-value* |
|---|---|---|---|---|
| OTP positive | 57 (72) | 16 (20) | 41 (52) | 0.001 |
| OTP negative | 22 (28) | 15 (19) | 7 (9) | |
| CD44 positive | 62 (78) | 19 (24) | 43 (54) | 0.003 |
| CD44 negative | 17 (22) | 12 (15) | 5 (6) | |
| Ki-67 <5% | 59 (75) | 15 (19) | 44 (56) | <0.001 |
| Ki-67 ≥5% | 20 (25) | 16 (20) | 4 (5) | |
| No distant metastasis | 72 (91) | 30 (38) | 42 (53) | 0.236 |
| Distant metastasis[ǂ] | 7 (9) | 1 (1) | 6 (8) | |

Table S3. Characteristics of patients with neutral biomarker profile (*n*=79); TC: typical carcinoid; AC: atypical carcinoid; ǂ occurrence of distant metastasis during follow up; * calculated using the Chi-Squared or Fisher's Exact test.
